# Patient navigation for lung cancer screening among current smokers in community health centers a randomized controlled trial

**DOI:** 10.1002/cam4.1297

**Published:** 2018-02-21

**Authors:** Sanja Percac‐Lima, Jeffrey M. Ashburner, Nancy A. Rigotti, Elyse R. Park, Yuchiao Chang, Salome Kuchukhidze, Steven J. Atlas

**Affiliations:** ^1^ Division of General Internal Medicine Massachusetts General Hospital Boston Massachusetts; ^2^ Harvard Medical School Boston Massachusetts; ^3^ Mongan Institute for Health Policy Massachusetts General Hospital Boston Massachusetts; ^4^ Massachusetts General Hospital Cancer Center Boston Massachusetts

**Keywords:** Cancer screening, health center, lung cancer, patient navigation, smokers

## Abstract

Annual chest computed tomography (CT) can decrease lung cancer mortality in high‐risk individuals. Patient navigation improves cancer screening rates in underserved populations. Randomized controlled trial was conducted from February 2016 to January 2017 to evaluate the impact of a patient navigation program on lung cancer screening (LCS) among current smokers in five community health centers (CHCs) affiliated with an academic primary care network. We randomized 1200 smokers aged 55–77 years to intervention (*n* = 400) or usual care (*n* = 800). Navigators contacted patients to determine LCS eligibility, introduce shared decision making about screening, schedule appointments with primary care physicians (PCPs), and help overcome barriers to obtaining screening and follow‐up. Control patients received usual care. The main outcome was the proportion of patients who had any chest CT. Secondary outcomes were the proportion of patients contacted, proportion receiving LCS CTs, screening results and number of lung cancers diagnosed. Of the 400 intervention patients, 335 were contacted and 76 refused participation. Of the 259 participants, 124 (48%) were ineligible for screening; 119 had smoked <30 pack‐years, and five had competing comorbidities. Among the 135 eligible participants in the intervention group, 124 (92%) had any chest CT performed. In intention‐to‐treat analyses, 124 intervention patients (31%) had any chest CT versus 138 control patients (17.3%, *P* < 0.001). LCS CTs were performed in 94 intervention patients (23.5%) versus 69 controls (8.6%, *P* < 0.001). A total of 20% of screened patients required follow‐up. Lung cancer was diagnosed in eight intervention (2%) and four control (0.5%) patients. A patient navigation program implemented in CHCs significantly increased LCS among high‐risk current smokers.

## Introduction

Lung cancer is the leading cause of cancer death among men and women in the United States. An estimated 220,500 new cases of lung cancer are expected in 2017 along with 155,870 lung cancer related deaths 1. The majority of individuals with lung cancer are diagnosed at an advanced stage when cure is no longer an option 2. This is particularly true for underserved populations where low socioeconomic status and inadequate access to care contribute to inequities in lung cancer care and higher rates of morbidity and mortality 3, 4, 5, 6, 7.

In 2011, the National Lung Screening Trial (NLST) showed that lung cancers could be detected earlier and, thus, lung cancer mortality could be decreased with annual screening chest computed tomography (CT) 8, 9. In 2013, United States Preventive Task Force recommended yearly lung cancer screening for high‐risk individuals, and in 2015 Center for Medicare and Medicaid Services (CMS) issued requirements for coverage of screening for lung cancer 10, 11. In spite of insurance coverage for screening, a recently published study based on the National Health Interview Survey found less than 4% of eligible smokers received lung cancer screening in the previous year, and rates had not significantly changed from 2010 to 2015 12. It is uncertain what accounts for the slow uptake of lung cancer screening, but factors may include patients’ and provider's lack of knowledge about screening, patients’ access and desire to have lung screening, and system barriers 13, 14, 15, 16, 17, 18.

Patient navigation is a strategy demonstrated to improve cancer screening and follow‐up in underserved populations 19, 20, 21, 22, 23, 24. Patient navigators are culturally and linguistically tailored outreach workers who help patients overcome barriers to receiving the care they need 20, 25, 26. In the most vulnerable populations, those with low income and educational attainment and racial/ethnic minorities, patient navigation can improve cancer screening rates 27, 28, follow‐up after abnormal results 29, and decrease disparities in care 30.

The objective of our study was to develop and evaluate a patient navigation (PN) program to promote lung cancer screening among low socioeconomic status current smokers receiving primary care in community health centers affiliated with an academic primary care network.

## Methods

### Study setting

We conducted a randomized controlled trial from February 29, 2016 to January 31, 2017 at the five community health centers affiliated with Massachusetts General Hospital. The institutional review board approved all study activities and provided a waiver of written informed consent. Participants were not compensated.

### Study population and randomization

**Figure 1:**
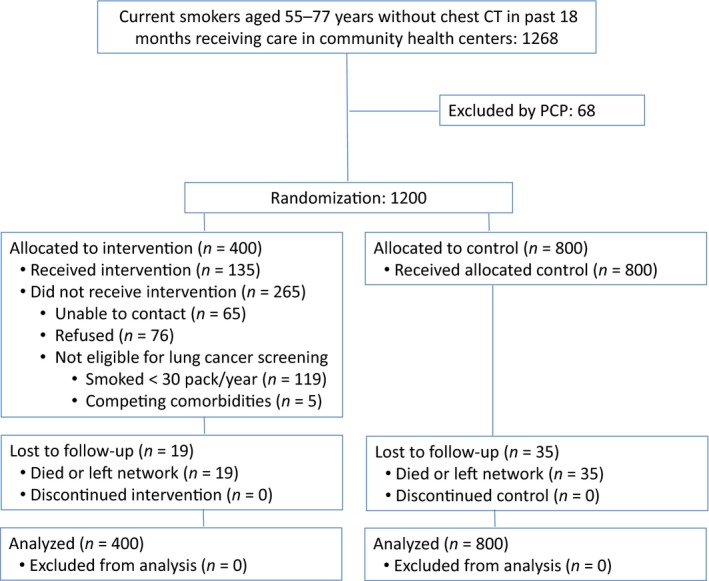
CONSORT diagram depicting the flow of study patients through randomization, intervention, and outcome analysis.

Using the primary care practice network's existing population health management information technology system 27, 31, 32, we developed a registry to identify current smokers who might be eligible for lung cancer screening. Eligibility criteria included patients aged 55–77 years old who were identified as current smokers in the electronic medical record (EMR). Since the majority of our community health center patients have public insurance, we used the upper age criteria set by CMS 10. We excluded patients who had any chest CT performed in the previous 18 months and those not receiving care in one of the five community health centers. As shown in Figure 1, we identified 1268 potentially eligible patients. Primary care providers (PCPs) were given an opportunity to review their patient list and excluded 68 patients because of competing comorbidities (8), insufficient smoking history (15), death (1), moved/not my patient (6), or other unstated reason (38). The remaining 1200 patients were randomized in a 1:2 ratio to patient navigation (*n* = 400) or usual care (*n* = 800) stratified by PCP. The 400 intervention patients were estimated to be the maximum number of individuals who could be navigated during the study period with available resources.

### Intervention

Prior to the start of the study, the principal investigator introduced the program and provided an educational session about lung cancer screening at PCP team meetings in community health centers. Content included patient eligibility criteria, Medicare reimbursement requirements, how to perform shared decision making (SDM), and order the lung cancer screening CT in our system.

Four part‐time lay patient navigators with bachelor's degrees were trained in motivational interviewing, problem solving, goal setting, use of the IT system, and electronic medical record documentation. Specifically for this program, navigators were educated about lung cancer and screening with low‐dose CTs. Navigator activities included performing an initial interview with patients to determine smoking history/eligibility for lung cancer screening, identifying barriers and working with patients to overcome barriers to screening, introducing SDM, and empowering patients to discuss the risks and benefits of screening with their PCP. Navigators learned how to review CT lung cancer screening reports and to communicate abnormal results to the ordering provider and arrange for appropriate follow‐up. As part of their training in smoking cessation, all navigators completed an online course developed by the University of Massachusetts Medical School Center for Tobacco Treatment Research and Training, “Basic Skills Training for Working with Smokers.” Navigator training lasted about 2 weeks depending on their previous experience. A procedure manual was created and available to the navigation team to ensure outreach consistency. The manual provided phone scripts, talking points, time frames for outreach calls, and templates to facilitate documentation in the EMR and communication with PCP. The cancer care patient navigation team manager participated in training and provided daily supervision of LCS navigators. Each week the navigator team met with the principal investigator to discuss progress and challenges.

Initial patient contact in the intervention group consisted of a mailed letter about the program and educational materials. One week later, the navigator contacted patients by telephone, introduced the study, obtained a verbal assent to participate and, for patients interested in the program, took a detailed smoking history to determine if they were eligible for lung cancer screening (more than 30 pack‐year history) 10, 33. For eligible patients, the navigator reviewed the purpose of lung cancer screening and explored their concerns and barriers to screening. Both the risks of screening, such as exposure to radiation and false positive results, and the benefits were explained. If the patient was interested in screening, the navigator arranged a SDM appointment with their PCP so lung cancer screening could be discussed and ordered, if appropriate. Additional navigator interventions could involve reminding the patient about the scheduled CT screening test, helping with translation, insurance issues, transportation, and overcoming other system barriers as needed. Outreach to patients was mostly via phone calls.

Navigators also provided brief smoking cessation counseling. If interested in quitting, smokers were connected to existing smoking cessation resources. The navigator could place a referral to the Massachusetts Quitline or to a tobacco cessation specialist working in the community health centers. If the patient was interested in smoking cessation medication, the navigator would notify the provider.

After a patient completed LCS, the navigator reviewed the CT results in the EMR. If the patient had an abnormal test result that required follow‐up, the navigator contacted the patient's PCP and helped the patient obtain timely follow‐up.

The navigators used the population health management IT registry tool to follow patients randomized to the intervention group and to track lung CT tests scheduled and completed. They also recorded each patient contact and documented interventions performed. Actions taken at SDM visits with the PCP were documented in the electronic health record.

Patients randomized to the control arm received usual care. Prior to the study start, all PCPs were educated about screening guidelines and procedures to order screening CT so they could offer SDM and order lung cancer screening for all their patients including the usual care group. PCPs in the usual care group could identify their eligible patients, discuss screening at a SDM visit and order a screening CT, but without support from PNs.

### Study outcomes

The primary outcome was the proportion of patients in intervention and control groups who had a chest CT performed for screening or diagnostic purposes during the study period. Secondary outcomes included the proportion of patients receiving lung cancer screening CTs, results of screening CTs, and lung cancer diagnoses in the intervention and control groups.

Patient characteristics and prior chest CT data were obtained from the EMR. Dates and type of CT scans were obtained from electronic reports or billing data.

Radiologists reviewing lung cancer screening CTs reported findings using Lung CT Screening Reporting and Data System (Lung‐RADS) as recommended by the American College of Radiology 34. Medical records of patients with Lung‐RADS 3 and 4 were reviewed to identify additional imaging and procedures performed and their results. We calculated the time from the screening CT to radiologist recommended follow‐up. The stage and outcomes of lung cancer diagnosed with screening and diagnostic CTs were assessed.

### Statistical analyses

An intention to treat analysis was used for the primary outcome and included all 1200 randomly assigned patients. We used the generalized estimating equations (GEE) approach to take into account the clustering of patients by PCP. Logistic regression models with GEE were used to compare proportions between intervention and control groups. We also explored between‐group differences in completion of any chest CT and screening CTs during the study period in intervention and control groups in relevant subgroups defined by race, primary language, insurance, gender, and age.

Among patients who had a lung cancer screening CT, we compared the distribution of CT findings between intervention and control groups using a chi‐square test. We hypothesized that the navigator program would increase lung cancer screening in the intervention group by at least 10%. With a total sample size of 1200 smokers including 400 randomized to the intervention group and an alpha level of 0.05, the sample provided 80% power to detect a 10% difference after accounting for clustering by provider.

Two‐sided significance tests with *P*‐values < 0.05 were considered statistically significant. All statistical analyses were performed using a commercial software package (PROC GENMOD, SAS version 9.4; SAS Institute, Cary, NC).

## Results

At baseline, 400 patients were randomized to the intervention arm, and 800 patients were randomized to the control arm (Fig. 1). The average age of participants was 62.3 years (SD: 5.6), 52.5% were women, 81.4% white, 88.8% spoke English, and 55.0% were insured through Medicaid. About two‐thirds (64.7%) finished only high school or less. The sociodemographic characteristics were similar between intervention and control groups (Table 1).

**Table 1:** Baseline characteristics among 1200 intervention and control patients

Characteristics	Intervention (*n* = 400)	Control (*n* = 800)
Age, mean (SD)	61.8 (5.4)	62.4 (5.7)
Clinic visits over 3 years, mean (SD)	10.3 (7.9)	10.7 (8.4)
Gender, female	188 (47.0%)	442 (55.3%)
Race		
Asian	18 (4.5%)	22 (2.8%)
Black	18 (4.5%)	25 (3.1%)
Hispanic	26 (6.5%)	41 (5.1%)
Other/Unknown	27 (6.8%)	46 (5.8%)
White	311 (77.8%)	666 (83.3%)
Language, English	352 (88.0%)	714 (89.3%)
Insurance		
Commercial	128 (32.0%)	280 (35.0%)
Medicare	47 (11.8%)	84 (10.5%)
Medicaid	121 (30.3%)	211 (26.4%)
Dual Medicare/Medicaid	103 (25.8%)	225 (28.1%)
Self	1 (0.3%)	0 (0.0%)
Education		
Less than high school	60 (15.0%)	120 (15.0%)
Graduated high school or GED	204 (51.0)	382 (47.8%)
Some college/vocational program	43 (10.8%)	119 (14.9%)
College/graduate school	76 (19.0%)	138 (17.3%)
Other/Unknown	17 (4.3%)	41 (5.1%)

Among intervention patients, 335 (83%) were successfully contacted and 76 refused participation. Of the 259 participating patients, 124 (48%) were not eligible for lung cancer screening; 119 (46%) had smoked <30 pack‐years, and 5 (2%) had competing comorbidities (Fig. 1). Among the 135 patients eligible for lung cancer screening in the intervention group, 124 (92%) had a diagnostic or screening chest CT during the study period.

Navigators were able to assess eligibility for lung cancer screening in 259 patients (65%). Navigator interventions provided to 135 eligible patients in the intervention group included: introduce SDM (53%), schedule screening CT (64%), and provide reminders about the test (47%) (Table 2). For 77% of patients, navigators emailed or messaged PCPs regarding upcoming SDM appointment and/or to order a follow‐up CT. Almost all patients (98%) received brief smoking counseling and interested patients (49%) were referred to tobacco cessation resources.

**Table 2:** Navigators’ interventions for patients eligible for lung cancer screening in the intervention group

Intervention	*N* (135)	%
Chart review	133	98.5
Patient education/motivation	41	30.4
Addressing barriers to screening	23	17.0
Introduce shared decision making	72	53.3
E‐mail/message provider	104	77.0
Scheduled LSC CT	87	64.4
Patient reminder about LCS CT	64	47.4
Brief smoking counseling	133	98.5
Referred to cessation resources	66	48.9

**Figure 2:**
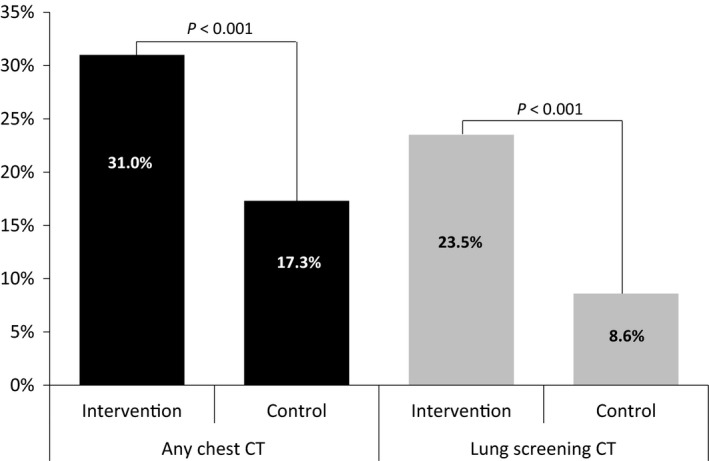
Proportion of all chest CTs and lung cancer screening CTs in intervention and control group (Intention‐to‐treat analyses).

**Figure 3:**
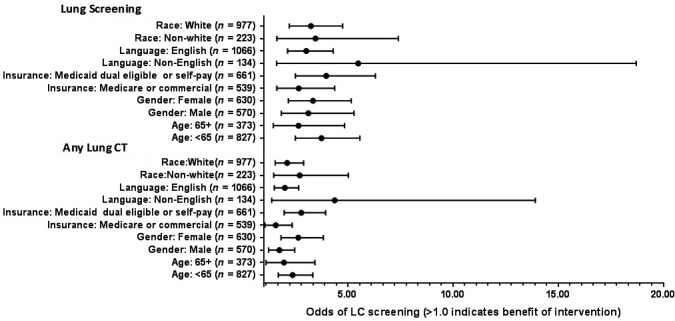
Odds Ratios and 95% confidence intervals for lung cancer screening in intervention and comparison groups stratified by patient subgroups.

The primary outcome, any chest CT performed during the study period, was compared using an intention‐to‐treat analysis (Fig. 2). During the study period, a greater proportion of patients in the intervention group had any chest CT compared to patients in the control group (31.0% [124] vs. 17.3% [138], *P* < 0.001). Similarly, lung cancer screening CTs were performed more often in the intervention group compared to the control group (23.5% [94] vs. 8.6% [69], *P* < 0.001). Exploratory analyses examined the effect of the navigator program stratified by different subgroups and found a beneficial effect in all subgroups (Figure 3).

Among patients who had a lung cancer screening CT, the distribution of Lung‐RADS findings was similar between the two groups (*P* = 0.72, Table 3). In both groups, the majority of patients had Lung‐RADS 1 or 2 findings and did not require further follow‐up (intervention 79.8% and control 82.6%). In the intervention group, 12 (12.8%) patients had Lung‐RADS 3 findings and required a six–month follow up compared to 6 (8.7%) in the control group. Seven (7.4%) in the intervention group and 6 (9.6%) in control patients had Lung‐RADS 4 findings and required immediate follow‐up.

**Table 3:** Lung cancer screening CT results in intervention and usual care group

Lung‐RADs score	Intervention (*n* = 94)	Usual care (*n* = 69)	*P* value
1	19 (20.2%)	18 (26.1%)	
2	56 (59.6%)	39 (56.5%)	0.72
3	12 (12.7%)	6 (8.7%)	
4	7 (7.4%)	6 (8.7%)	

Within the study period, 3 of 4 patients in the intervention group with Lung‐RADS 3 findings had recommended 6‐month follow‐up. All 13 patients who had a Lung‐RADS 4 finding received follow‐up (average time 20.1 days in intervention and 22.0 days in control). The number of additional diagnostic tests post‐screening was similar in both groups: in the navigated group 2 patients had a PET CT, 3 repeat chest CT, 1 an abdominal CT, 1 brain MRI, and 1 patient had a mediastinoscopic biopsy. Among screened patients in the usual care group 4 had a PET CT, 5 repeat chest CT, and 1 an abdominal CT.

Eight lung cancers were diagnosed in intervention patients (2%) compared to 4 in control patients (0.5%). Three patients (2 in the intervention group and 1 in the control group) were diagnosed with lung cancer after a screening CT and had surgical resection. One patient with stage 1 disease had only a surgical resection. Two patients with stage 3 disease received surgery followed by chemotherapy and chemotherapy with radiation. Six of nine cancers identified after a diagnostic chest CT were stage 4, and 3 patients died. Additionally, two patients with stage 1 disease could not have surgery due to the presence of serious comorbid conditions.

## Discussion

This study evaluated the impact of a patient navigation program for lung cancer screening among current smokers receiving care in community health centers. Though many patients in the intervention group did not meet pack‐year eligibility criteria for screening, almost all navigated patients eligible for and interested in lung cancer screening had a chest CT during the study period. Patients randomized to the PN intervention had significantly higher rates of receiving a screening or diagnostic chest CT compared to patients receiving usual care over the 11‐month study period. This difference was mostly due to patients in the PN intervention arm having nearly a threefold higher rate of lung cancer screening CTs compared to patients receiving usual care.

To the best of our knowledge, this is the first study using lay navigators to help patients cared for in community health centers receive lung cancer screening. Navigators took multiple actions to facilitate the complex process of initiating lung cancer screening for patients and their providers 10. As in other cancer screening PN programs, navigators educated, motivated, and helped patients overcome obstacles to get screened 27, 28. For lung cancer screening, navigators also helped PCPs by assessing patient eligibility, introducing smoking cessation and SDM to patients prior to a visit, and by reminding PCPs about upcoming SDM appointments and helping to follow‐up abnormal results. The PN program appeared efficacious in all patient subgroups examined, but further studies are needed to determine which navigator interventions were most important.

Kinsinger et al. 35 recently described lung cancer screening efforts in the Veterans Health Administration (VHA). Full time nurses or middle‐level health care professionals were used as screening coordinators who performed comparable actions as the trained lay navigators in this study. They encountered similar challenges in implementing their program including missing or inadequate smoking data in the EMR. This resulted in excluding two‐thirds of their initial cohort due to ineligibility because of smoking history. Since we did not include former smokers who may be expected to have lower rates of eligibility, our exclusion rate was lower, almost half of smokers identified. In our study navigators obtained a detailed smoking history and updated EMR for intervention patients. However, other less expensive and more generalizable ways to obtain and document smoking history are needed for lung cancer screening programs to be efficient for current and former smokers.

In this study, 20% of patients required follow‐up of abnormal findings. This is higher than observed in NLST (12.8%) but similar to VHA study (20%) when Lung‐RADS criteria were retrospectively applied 35, 36. Comparing abnormal rates using Lung‐RADS criteria among these studies suggest that patients in the VHA and our study may have been at higher risk than NLST participants. This is supported by a lower proportion of patients diagnosed with lung cancer in the NLST 7, compared to the VHA and our study as well as a report evaluating free lung cancer screening in an underserved southeastern US population 35, 37.

The percent of patients with abnormal chest CT finding is important because follow‐up rates are not optimal 38. The benefit of screening is only possible if there is a system to ensure timely follow‐up. Thus, in addition helping ensure completion of lung cancer screening, navigators coordinated follow‐up appointments and testing for abnormal results. We observed that patients with abnormal CT results in both groups received recommended follow‐up during the study period. In our study we used lay PNs known to be effective for other cancer screenings. The lay PNs have limited clinical knowledge. Still, we showed that these lay PNs can be very effective in improving lung cancer screening and cost was less than half of an oncology nurse navigator. However, the oncology nurse navigators as trained healthcare professional might provide better communication during abnormal screening follow‐up and lung cancer treatment 34. Larger studies with longer follow‐up are needed to evaluate the potential impact and cost effectiveness of lay PNs compared to oncology nurse navigators on follow‐up of abnormal lung cancer screening in underserved populations 39.

Our study has limitations. Results from five urban community health centers affiliated with an academic medical center, a population health IT infrastructure, and an established patient navigator program may not be generalizable to other clinical settings. Because we could not assess the proportion of current smokers in the control group who were eligible for lung cancer screening, we used intention‐to‐treat analyses and expect the patient‐level randomization resulted in similar numbers eligible in each group. Some patients might have obtained chest CTs at outside facilities that were not included in our analyses, but we would expect similar proportions in intervention and control groups. Since randomization occurred at the patient‐level, all PCPs received education about the study and procedures for SDM and ordering lung cancer screening. It is possible that this may have increased screening in the control population and decreased the magnitude of the observed intervention effect. Our eligibility criteria differ from the existing screening recommendations. Because CMS covers screening through age 77 10, we excluded patients 78–80 years old who are eligible according to the USPTF guidelines 11. We also excluded former smokers due to limitations of data in our EMR to identify those eligible for screening. We used NLST criteria and included participants who did not have a chest CT in the last 18 months. This enabled us to identify patients without a prior screening chest CT. The initial screening lung CT requires a SDM visit and PNs were trained to facilitate that process 10. Future studies need to explore the impact of patient navigation for LCS of former smokers and on USPTF recommended yearly follow‐up screening 11.

## Conclusion

Screening for lung cancer is challenging because it requires effort to identify eligible high‐risk individuals and engage patients and clinicians in shared decision making. Among current smokers aged 55–77 in community health centers, we demonstrated that those randomly assigned to a patient navigation program had higher rates of lung cancer screening compared to patients receiving usual care. In this program, navigators served as liaisons between patients and their primary care team to mitigate barriers to receiving care and helped almost all high‐risk smokers interested in lung cancer screening get screened. By increasing lung cancer screening rates in underserved and low‐income current smokers, navigation may improve equity in care while decreasing lung cancer mortality.

## Conflict of Interest

Massachusetts General Hospital entered into a royalty arrangement on June 27, 2013, to commercializethe population management system with SRG Technology, a for‐profit company. Dr. Atlas is a beneficiary of this royalty arrangement but has not received any payments to date. Dr. Atlas has received payments as a consultant for the company.
